# Personalized Treatment Selection and Disease Monitoring Using Circulating Tumor DNA Profiling in Real-World Cancer Patient Management

**DOI:** 10.3390/diagnostics10080550

**Published:** 2020-08-02

**Authors:** Julius Wehrle, Ulrike Philipp, Martina Jolic, Marie Follo, Saskia Hussung, Silvia Waldeck, Max Deuter, Michael Rassner, Jan Braune, Justyna Rawluk, Christine Greil, Cornelius F. Waller, Heiko Becker, Jesús Duque-Afonso, Anna L. Illert, Ralph M. Fritsch, Frank Meiss, Justus Duyster, Nikolas von Bubnoff, Florian Scherer

**Affiliations:** 1 Department Medicine I, Medical Center—University of Freiburg, Faculty of Medicine, University of Freiburg, 79106 Freiburg, Germany; julius.wehrle@uniklinik-freiburg.de (J.W.); ulrike.philipp@uniklinik-freiburg.de (U.P.); martina.jolic@uniklinik-freiburg.de (M.J.); marie.follo@uniklinik-freiburg.de (M.F.); saskia.hussung@usz.ch (S.H.); silvia.waldeck@uniklinik-freiburg.de (S.W.); max.deuter@uniklinik-freiburg.de (M.D.); michael.rassner@uniklinik-freiburg.de (M.R.); jan.braune@uniklinik-freiburg.de (J.B.); justyna.rawluk@uniklinik-freiburg.de (J.R.); christine.greil@uniklinik-freiburg.de (C.G.); cornelius.waller@uniklinik-freiburg.de (C.F.W.); heiko.becker@uniklinik-freiburg.de (H.B.); jesus.duque.afonso@uniklinik-freiburg.de (J.D.-A.); lena.illert@uniklinik-freiburg.de (A.L.I.); ralph.fritsch@usz.ch (R.M.F.); justus.duyster@uniklinik-freiburg.de (J.D.); 2 Department of Medical Oncology and Hematology, Zurich University Hospital, 8091 Zurich, Switzerland; 3 Department of Dermatology, Medical Center—University of Freiburg, Faculty of Medicine, University of Freiburg, 79104 Freiburg, Germany; frank.meiss@uniklinik-freiburg.de; 4 Department of Hematology and Oncology, University Hospital Schleswig-Holstein, Campus Lübeck, 23562 Lübeck, Germany

**Keywords:** circulating tumor DNA, liquid biopsy, digital-droplet PCR, noninvasive routine diagnostics, ctDNA-based treatment selection, prediction of cancer progression

## Abstract

Background: Circulating tumor DNA (ctDNA) in the blood plasma of cancer patients is an emerging biomarker used across oncology, facilitating noninvasive disease monitoring and genetic profiling at various disease milestones. Digital droplet PCR (ddPCR) technologies have demonstrated high sensitivity and specificity for robust ctDNA detection at relatively low costs. Yet, their value for ctDNA-based management of a broad population of cancer patients beyond clinical trials remains elusive. Methods: We developed mutation-specific ddPCR assays that were optimized for their use in real-world cancer management, covering 12 genetic aberrations in common cancer genes, such as *EGFR*, *BRAF*, *KIT*, *KRAS*, and *NRAS*. We assessed the limit of detection (LOD) and the limit of blank (LOB) for each assay and validated their performance for ctDNA detection using matched tumor sequencing. Results: We applied our custom ddPCR assays to 352 plasma samples from 96 patients with solid tumors. Mutation detection in plasma was highly concordant with tumor sequencing, demonstrating high sensitivity and specificity across all assays. In 20 cases, radiographic cancer progression was mirrored by an increase of ctDNA concentrations or the occurrence of novel mutations in plasma. Moreover, ctDNA profiling at diagnosis and during disease progression reflected personalized treatment selection through the identification of actionable gene targets in 20 cases. Conclusion: Collectively, our work highlights the potential of ctDNA assessment by sensitive ddPCR for accurate disease monitoring, robust identification of resistance mutations, and upfront treatment selection in patients with solid tumors. We envision an increasing future role for ctDNA profiling within personalized cancer management in daily clinical routine.

## 1. Introduction

The use of circulating tumor DNA (ctDNA) as a clinical biomarker has the potential to revolutionize the management of tumor patients. Cancer cells release fragments of ctDNA into the bloodstream, where they can be repeatedly sampled throughout the course of a disease (=liquid biopsy) [1]. Profiling of ctDNA facilitates robust and accurate noninvasive disease monitoring and characterization of tumor genotypes in most cancer types [2,3,4,5,6]. The role of ctDNA as a biopsy-free biomarker has been heavily investigated in several laboratory-based studies and clinical trials, particularly in patients with advanced solid cancers. The assessment of ctDNA has shown utility for several clinical applications, such as disease monitoring, the prediction of clinical outcomes, the identification of resistance mechanisms, and personalized treatment selection [4,5,7,8,9,10,11,12,13,14,15,16,17,18,19,20]. Yet, the value of ctDNA for routine management of a broad population of cancer patients beyond clinical trials remains elusive.

PCR-based platforms optimized for ctDNA assessment in plasma have been extensively studied in recent years, with variable performance depending on the assay type and target gene [21]. Digital-droplet PCR (ddPCR) is based on the dispersion of DNA molecules into thousands of nanoliter-sized droplets for subsequent PCR amplification and probe-based detection [22,23]. Due to its wide quantitative range, ddPCR combines high sensitivity and specificity with robustness at relatively low costs compared to next-generation sequencing (NGS) technologies. Digital droplet PCR has demonstrated higher sensitivities compared to other PCR-based approaches, such as the cobas^®^ platform, a test that has received FDA approval for ctDNA-based molecular assessment [12,24,25]. However, the role of sensitive ddPCR for broad application in clinical routine is still unclear. 

Here, we report the results of a study in which we developed a variety of ddPCR assays for their use in the daily care of patients with solid tumors and its utility for disease monitoring and personalized treatment selection in the real-world setting. 

## 2. Materials and Methods

### 2.1. Patients

Patients enrolled in this work underwent treatment for solid cancer at the University Medical Center Freiburg, Germany. All patient blood samples were collected and analyzed as part of clinical routine between December 2015 and July 2019. The retrospective study was approved by the Freiburg Ethics Committee (No. 49/20; approved on 25 February 2020) in accordance with the Declaration of Helsinki. All radiographic examinations, laboratory analyses, histopathological and genetic tumor tissue analyses (including tumor sequencing, see below) were performed as part of standard clinical care. All clinical decisions were made based on tumor histopathology, tumor genotyping, ctDNA analyses, and clinical or radiographic assessment at the discretion of the treating physicians and in accordance with institutional standards and international guidelines. Blood samples from 20 healthy controls without suspected cancer were used for assay development and validation (see below).

### 2.2. Sample Collection and Processing

Peripheral blood samples (*n* = 352) were collected from 96 patients at various time points throughout their disease (Table 1 and Supplementary Table S1). Blood was drawn in 10 mL K_2_EDTA tubes and subsequently processed within 3 h to avoid contamination with cellular DNA from blood cell lysis. EDTA blood was centrifuged at 800× *g* for 10 min and the plasma compartment was separated and then centrifuged again at 1000× *g* for 10 min. Finally, the plasma was aliquoted into 2 mL tubes and stored at −80 °C. Cell-free DNA (cfDNA) was extracted from a median of 1.75 mL of plasma using the QIAsymphomy^®^ workflow (Qiagen, Hilden Germany) according to the manufacturer’s instructions. After isolation, cfDNA was quantified using the Qubit™ dsDNA High Sensitivity Kit (Life Technologies, Carlsbad, CA, USA). Isolated DNA was stored at −20 °C. Plasma samples from healthy controls were processed in the same manner. 

### 2.3. Digital Droplet PCR (ddPCR)

We designed and validated ddPCR assays for the detection of 10 known genetic aberrations in solid cancer. Two additional assays were obtained from Bio-Rad Laboratories (Feldkirchen, Germany) and independently validated in our laboratory (Supplementary Figure S1A). Locked nucleic acid (LNA) probes and corresponding primer pairs were designed using the Beacon Designer v.8.20 software (Premier Biosoft, Palo Alto, CA, USA). Primers and probes were manufactured by Integrated DNA Technologies (IDT, Leuven, Belgium). Mutant (Mut) probes were labeled with 6-carboxyfluorescein (FAM), wildtype probes were labeled with hexachlorofluorescein (HEX). Primer and probe sequences are listed in Supplementary Figure S1A. For each ddPCR run, 7 μL of the DNA template, 1.1 μL of the probes (250 nM final concentration each), and 0.22 μL of the primers (forward and reverse, 900 nM final concentration each) were added to 11 μL of ddPCR Supermix for Probes (Bio-Rad; total of 22 μL). Next, 20 μL of this mixture was automatically transferred into a cartridge of an Automated Droplet Generator (QX100/200^TM^, Bio-Rad) along with 70 μL of generation oil. After the droplets were generated, the reactions were transferred into a 96-well PCR plate (Bio-Rad). PCR was performed using a C1000 Touch thermal cycler (Bio-Rad). Droplet fluorescence was then measured using a QX100/200^TM^ Droplet Reader (Bio-Rad) and the data were analyzed using QuantaSoftware v1.7.4.0917 (Bio-Rad). For each assay, optimal PCR conditions and temperature gradients were determined (Supplementary Figure S1B). Within each run, one negative control (wildtype only), one positive control, and one without a template control were included.

### 2.4. ddPCR Assay Development

To determine limit of detection (LOD) for each assay, serial dilutions of the respective recombinant mutant DNA fragments (gBlock, IDT, USA) in the presence of human genomic DNA (Roche Diagnostics, Mannheim, Germany) were performed, while the relative mutant allele concentrations were set to 1:20, 1:40, 1:100, 1:200, 1:400, 1:800, 1:1000, 1:2000, and 1:10,000. Negative controls from healthy donors were included to assess false-positive droplets (background). Significant ctDNA detection was determined by comparing mutant DNA samples with the background using an unpaired two-tailed *t*-test. The LOD was calculated as the ratio of mutant copies vs. the total number of copies from the highest dilution that was significantly above the background (Supplementary Figure S2A). To determine limit of blank (LOB), ddPCR assays were run using cfDNA extracted from 10 healthy donors. The LOB was calculated as follows: LOB = mean_HealthyControl_ + 3 × (SD_Mean_) (Supplementary Figure S2B) [26]. A second independent cohort of negative controls from healthy donors (*n* = 10) was used to assess assay specificity (Supplementary Figure S3). An overview of the LODs, LOBs, and specificities for all 12 assays is provided in Supplementary Table S2.

### 2.5. Tumor Sequencing, Clinical Outcome Assessment, and Response Evaluation

Targeted NGS of tumor samples was performed as part of the clinical routine and according to the institutional guidelines at the Department of Pathology, University Medical Center Freiburg, Germany. In general, tumor genotypes were evaluated in advanced stage III and IV cancers, which was the majority of our cohort (83.3%, Table 1). Treatment response was assessed clinically and radiographically using either a CT scan, MRI, or PET-CT. Radiographic response was determined using the international Response Evaluation Criteria in Solid Tumors (RECIST) as part of standard clinical care [27]. 

### 2.6. Statistics

An unpaired two-tailed *t*-test was used to compare mutant DNA samples with the background to determine the LOD of all ddPCR assays (see above). *P*-values < 0.05 were considered significant. The concentration of ctDNA was expressed as the number of mutated cfDNA molecules per mL of plasma (mL plasma/well divided by the number of mutant copies/well = mutant copies/mL plasma). The mutant allele frequencies were expressed as a percentage (%) and were calculated by dividing the number of mutated cfDNA molecules per milliliter of plasma by the number of total analyzed cfDNA molecules per milliliter of plasma (i.e., mutant and wildtype cfDNA molecules), then multiplied by 100. Statistical tests were performed using GraphPad Prism (version 8.2.1) and MedCalc.

## 3. Results

### 3.1. ddPCR Assay Performance and Patient Characteristics

We individually designed and validated single-target ddPCR assays covering 12 hotspot mutations in *EGFR, BRAF, KRAS, NRAS,* and *KIT* genes, as described in the Materials and Methods section (Supplementary Figures S2 and S3). The probe and primer sequences and assay conditions are detailed in Supplementary Figure S1. The calculated LOD was in median 1:2145 (0.047%) and ranged between 1:753 (0.13%) and 1:9041 (0.011%) [18,28,29]. The LOB for each of the 12 assays is shown in Supplementary Table S2 and ranged from 0 to 17.8 mutated droplets per milliliter of plasma. To further assess the assay specificity and sensitivity, we applied all 12 assays to various independent sets of cfDNA samples. First, we analyzed cfDNA from an independent cohort of 10 healthy donors with each individual assay (Supplementary Figure S3). None of the 120 tests showed a positive result, demonstrating 100% specificity (Supplementary Table S2). Next, we profiled cfDNA from patients with available genotyping results from matched tumor biopsies, which served as gold standard (*n* = 56, Supplementary Figure S4). In 14/16 cases, tumor mutations were detectable in matched plasma samples, showing an overall sensitivity of 87.5%. On the other hand, negative tumor genotyping results were confirmed by ctDNA profiling in 38/40 cases (95% specificity, Supplementary Figure S4A). In two patients with non-small cell lung cancer (NSCLC), *EGFR* T790M mutations were identified in the plasma, while tumor biopsies tested negative for this genetic aberration. In both cases, the mutation emerged at radiographic disease progression during first- or second-generation EGFR inhibitor therapy. Moreover, *EGFR* T790M was detectable in a prior tumor sample from patient FR069, suggesting that spatial tumor heterogeneity and subclonal events were missed by tumor sequencing, yet captured by ctDNA analyses of matched plasma samples (Supplementary Figure S4B,C). 

Having demonstrated the technical performance of the assays, we next applied them in the real-world setting to 352 plasma samples from 96 patients with solid tumors over a period of 3.5 years from December 2015 until July 2019. The patient and sample characteristics are summarized in Table 1 and Supplementary Table S1. The median age was 66 years (range: 27–91 years) and 55.2% of patients were female. More than 90% of patients were diagnosed with either NSCLC (56/96, 58.3%) or melanoma (31/96, 32.3%), and 62.5% had metastatic stage IV disease (Table 1A). We performed a median of 3.7 tests per patient, with a median input plasma volume of 1.75 mL. The most frequently found genetic aberrations were *EGFR* T790M (*n* = 152, 43.4%), followed by *EGFR* Del19 (*n* = 52, 14.6%), *EGFR* L858R (*n* = 52, 14.6%), and *BRAF* V600E (*n* = 44, 12.6%, Table 1B). 

### 3.2. Prediction and Detection of Disease Progression Using ctDNA Monitoring

**Figure 1:**
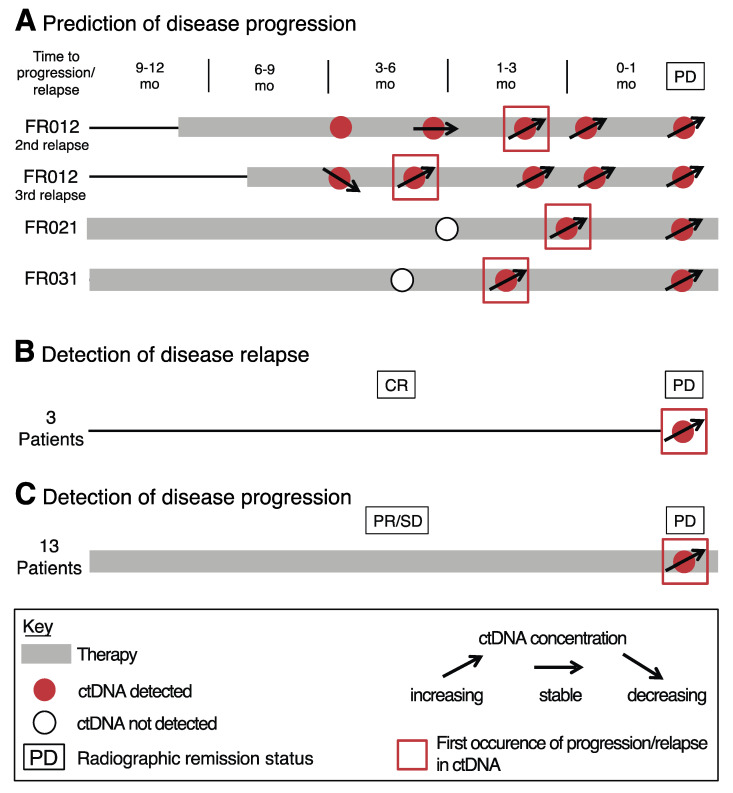
Disease progression mirrored by ctDNA concentrations. (**A**) Patient data demonstrating ctDNA detection before radiographic disease progression. (**B**,**C**) Accumulative patient data showing ctDNA detection at disease relapse after a complete response (**B**) or an increase of ctDNA levels at disease progression (**C**). Open circle, ctDNA not detected; red circle, ctDNA detected; black ascending arrow, increasing ctDNA levels; black descending arrow, decreasing ctDNA levels; black horizontal arrow, stable ctDNA levels; red square, first appearance of ctDNA increase before radiographic disease progression; black rectangle, radiographic remission status; grey bar, treatment; CR, complete response; ctDNA, circulating tumor DNA; mo, months; PD, progressive disease; PR, partial response; SD, stable disease.

Noninvasive monitoring of treatment response and cancer progression is among the most promising clinical applications of ctDNA as a biomarker. Therefore, we profiled available plasma samples from a subset of 19 patients, all of whom experienced radiographic disease progression during cancer treatment or recurrence after a complete response (CR). We identified increasing ctDNA levels or emerging mutations by ddPCR in all 19 patients, reflecting tumor growth or the occurrence of novel cancer subclones (Figure 1 and Supplementary Figure S5). While increasing ctDNA concentrations or novel aberrations were detected in all patients at the time of radiographic progression (Figure 1B,C and Supplementary Figure S5B,C), ctDNA was also detected as minimal residual disease (MRD) before radiographic progression in at least one plasma sample from four different cases, with ctDNA levels as low as 3.25 mutant copies per mL of plasma (0.17% allele frequency (AF), FR031) (Figure 1A and Supplementary Figure S5A). Here, the time between the first increase of ctDNA concentrations and clinical progression ranged from 34 to 130 days (Figure 1A and Supplementary Figure S5A). On the other hand, ctDNA was undetectable in plasma samples obtained from seven patients who remained in CR after cancer therapy, again demonstrating high specificity (not shown). 

### 3.3. Identification of Mutations in Actionable Gene Targets at Cancer Diagnosis

**Figure 2:**
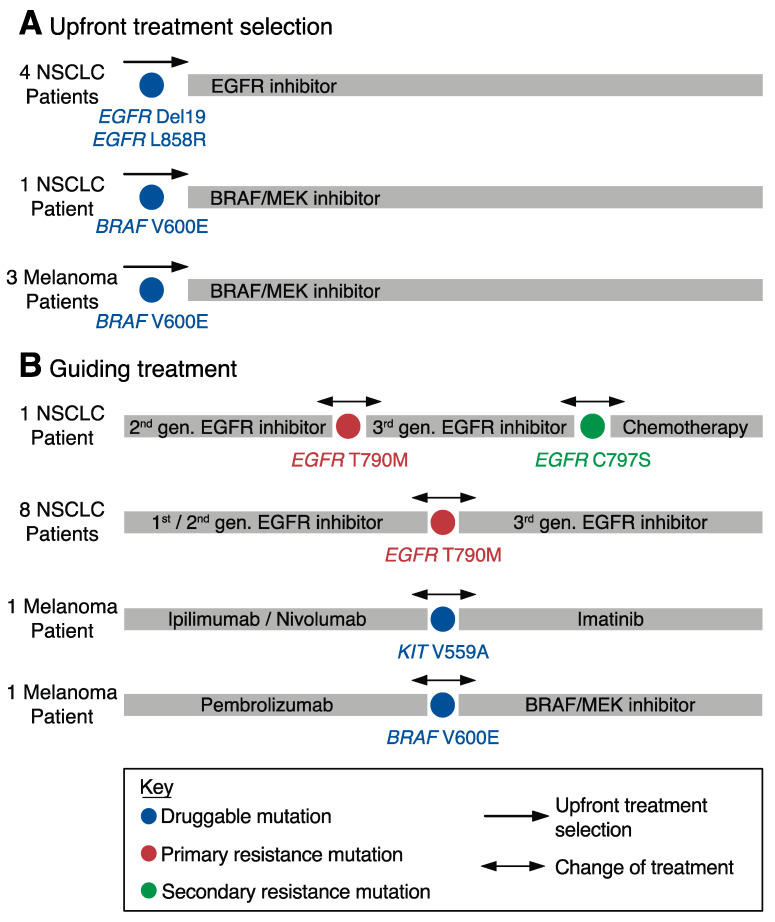
Therapy selection reflected by ctDNA profiling. (**A**) Patient data demonstrating upfront treatment selection in eight patients, which was reflected by the identification of mutations in ctDNA. (**B**) Detection of resistance mutations in blood plasma using ctDNA profiling in 11 patients. Black arrows indicate treatment selection or change of therapy based on mutation detection in actionable gene targets (colored in blue, red, and green). 1st, first; 2nd, second; 3rd, third; gen., generation. NSCLC, non-small cell lung cancer.

In the era of personalized medicine, characterizing the genetic landscape and identifying actionable mutations in treatment-naïve metastatic patients has become essential in clinical practice to allow clinicians to select patients who are most likely to benefit from targeted therapies. If tumor biopsies are scarce or unavailable, or when the patient’s health condition does not allow for invasive procedures, profiling of ctDNA can be considered for the detection of drug targets [30]. We identified nine patients with advanced tumors in our cohort (six NSCLC and three melanoma) in whom the identification of genetic aberrations facilitated upfront treatment selection, and in whom plasma samples were available from the same time point (Figure 2A and Supplementary Figure S6). Tumor mutations were detectable in matched plasma samples from seven cases. In one additional patient with NSCLC (FL096), tumor material was very scarce and insufficient for tumor genotyping. Thus, we profiled three *EGFR* hotspot mutations (*EGFR* Del19, *EGFR* L858R, and *EGFR* T790M) in pretreatment plasma samples using ddPCR and identified a deletion in *EGFR* exon 19 that led to the initiation of therapy with the third-generation EGFR tyrosine kinase inhibitor (TKI) osimertinib (Supplementary Figure S6A, FL096). While one NSCLC patient was treated with combined BRAF and MEK inhibition due to the identification of a *BRAF* V600E mutation, four patients with NSCLC showed an aberration in *EGFR* (either *EGFR* Del19 or *EGFR* L858R) and were treated with osimertinib (Figure 2A and Supplementary Figure S6A). For melanoma, we always profiled three different *BRAF* hotspot mutations in parallel (i.e., *BRAF* V600E, *BRAF* V600K, and *BRAF* V600R) and found two *BRAF* V600E and one *BRAF* V600K mutation in three patients who did not receive any systemic treatment at testing. While one patient’s limited performance status did not allow for any further treatment (FR025), the other two patients obtained BRAF/MEK inhibitor therapy (Figure 2A and Supplementary Figure S6B).

Finally, an *EGFR* Del19 aberration was found by tumor sequencing at diagnosis in one patient (FR068) but was missed by ctDNA assessment (not shown), demonstrating the limitations of ctDNA profiling and the possibility of false-negative results [24].

### 3.4. Guiding Treatment through ctDNA Profiling

Most malignancies are characterized by remarkable molecular heterogeneity over time. During cancer therapy, temporal tumor heterogeneity has the potential to drive the evolution of molecular subclones that harbor critical resistance mechanisms [31]. Capturing these emerging subclones and characterizing molecular resistance is crucial during individualized cancer treatment to allow treating physicians to adapt their therapeutic strategies. The assessment of ctDNA is a promising approach for the detection of resistance mechanisms over time, as it is noninvasively accessible and repeatedly applicable during treatment. Here, we profiled ctDNA at the time of radiographic disease progression during systemic therapy and identified nine NSCLC and two melanoma patients in whom the detection of an emerging mutation guided further clinical management (Figure 2B and Supplementary Figure S7). All nine patients with NSCLC received first- or second-generation EGFR TKIs in the first-line setting. Clinical or radiographic progression was mirrored by the emergence of *EGFR* T790M mutations in the blood plasma in all nine cases. Consequently, eight patients received the third-generation TKI osimertinib to overcome the resistance, while one patient could not obtain osimertinib due to QT prolongation in the electrocardiogram (FR029, Figure 2B and Supplementary Figure S7A). Importantly, in six patients (66.7%), tumor material was not available due to inaccessibility or the patient’s poor performance status, hampering invasive tumor biopsies; thus, the identification of resistance was solely based on ctDNA assessment (FR012, FR015, FR021, FR029, FR044, FR066, and FR070). One of the osimertinib-treated patients (FR012), who initially responded to third-generation TKI, later acquired another emerging subclone that harbored *EGFR* C797S, which is a resistance mutation frequently observed in patients treated with osimertinib (Supplementary Figure S7A) [13]. 

Both melanoma patients acquired actionable mutations during therapy with checkpoint inhibitors that led to a change in treatment strategy. While one patient showed a *KIT* V559A mutation and was treated with imatinib, the other patient received BRAF/MEK inhibitors after the occurrence of *BRAF* V600E (Figure 2B and Supplementary Figure S7B). 

Collectively, these results highlight the importance of noninvasive ctDNA profiling during systemic treatment in real-world cancer management to identify relevant tumor heterogeneity that would otherwise be missed due to the lack of repetitive tumor biopsies.

## 4. Discussion

The clinical use of analytical tests to assess genomic variants in ctDNA has increased considerably in recent years. Various studies in advanced solid cancer showed a high concordance between variants detected in tumor biopsies and blood plasma [12,32,33,34]. Moreover, several trials have demonstrated the potential clinical utility for noninvasive detection of emerging resistance mutations during targeted therapies or the identification of actionable mutations in treatment-naïve patients, particularly in NSCLC [4,9,10,12,13,16,25,35,36,37]. Here, we present the design and validation of ddPCR assays for the detection of 12 hotspot mutations in ctDNA and their clinical utility for real-world routine cancer management. Digital droplet PCR is frequently used in laboratories for ctDNA assessment due to its robustness, precision, relatively low costs, and straightforward data analysis, allowing for accurate identification and monitoring of a single or a few genetic targets [18,23,28,29]. The limit of detection across all assays in our study was low, with a median LOD of 0.047%, enabling robust and sensitive ctDNA profiling. The technical validation revealed no false-positive results (100% specificity) and very high concordance with tumor tissue sequencing. Our overall patient sample specificity was 95% and sensitivity was 87.5%, which is higher than what was reported for qPCR assays, such as the cobas^®^ platform [12,24,25]. However, we observed four discrepant results between matched tumors and plasma that highlight the dilemma of interpreting results from ctDNA profiling. In two cases, actionable mutations identified in tumor biopsies from stage IV NSCLC patients were not detected in blood plasma. This demonstrates that it is crucial for physicians to know that not all tumors shed sufficient amounts of DNA into the bloodstream and that ctDNA assessment can produce false-negative results in a substantial number of cases. Therefore, a negative ctDNA result requires confirmation using tumor genotyping if possible [24]. In two other patients, *EGFR* T790M mutations were found in plasma but not in the matched tumor tissue. Here, tumor heterogeneity might represent a potential challenge to tissue-based genotyping, as ctDNA usually represents all tumor sites in the body, while genetic aberrations can be missed in a single tumor lesion [38]. In both cases, the clinical course and previous genetic findings suggest that both *EGFR* T790M mutations emerged in metastatic lesions that were not captured by tumor biopsies, illustrating the potential of ctDNA for the characterization of tumor heterogeneity [39].

We identified 19 patients from our cohort in whom an increase of ctDNA levels over time and the detection of ctDNA after CR predicted or reflected tumor progression. Correlations between changes in ctDNA concentrations and treatment responses or tumor progression have been shown in a large variety of studies across different types of cancer, including lung cancer and melanoma [3,9,16,18,29,40,41]. This potentially creates the opportunity for clinicians to change treatment even before clinical progression, not only in the setting of targeted therapies but also in patients receiving chemotherapy or immunotherapy. However, there is currently no evidence that changes in ctDNA levels and the early adaption of therapies have led to improved patient outcomes [42]. Our real-world data demonstrate that an increase of ctDNA levels coincides with radiographic disease progression in the majority of cases (16/19, 84%), while ctDNA analysis predicted subsequent disease progression in only three patients. Therefore, large prospective studies are needed to prove the clinical utility of ctDNA monitoring and the subsequent adaption of treatment paradigms.

In a total of 21 patients (21/96, 22%), ctDNA assessment using ddPCR revealed actionable mutations that contributed to the initiation of targeted therapies in treatment-naïve patients or to a change in therapeutic strategies due to the occurrence of resistance aberrations during systemic treatment. The vast majority of those mutations were identified in NSCLC patients and located in *EGFR* genes. However, actionable mutations were also identified beyond NSCLC in patients with melanoma. Here, four patients received BRAF/MEK inhibitors due to the detection of *BRAF* V600 mutations. The emergence of *EGFR* T790M mutations as a resistance mechanism to first- or second-line EGFR TKIs was observed in nine patients with NSCLC, resulting in the initiation of the third-generation TKI osimertinib. Of note, in the majority of these cases, change of therapy was solely based on a ctDNA assessment, as tumor material was not available or insufficient, demonstrating its clinical value in routine management. 

With third-generation TKIs being approved in the first-line setting of NSCLC patients with activating *EGFR* mutations, monitoring of *EGFR* T790M has become less important. However, the vast majority of NSCLC patients receiving third-generation EGFR TKIs either do not respond to therapy or ultimately undergo disease progression, suggesting that additional resistance mechanisms are decreasing the efficacy of these inhibitors [4,43]. In particular, the emergence of *EGFR* C797S mutations was observed in a large fraction of osimertinib-treated patients, which is an aberration that we also found in one patient from our cohort (FR012) [13]. Yet, resistance mechanisms to third-generation EGFR TKIs seem to be more heterogeneous, illustrating the limitations of PCR-based methods compared to NGS approaches, which generally cover larger regions of the genome and capture a broader variety of potential genetic targets [4]. 

Our real-world study was inherently limited by the heterogeneity of the patient cohorts and treatment strategies. Furthermore, blood samples were collected at the discretion of the treating physician in a routine clinical setting but not in a standardized way at various landmarks, hampering statistical analyses.

In summary, our work highlights the potential and clinical utility of ctDNA assessment using sensitive ddPCR in the routine management of patients with solid tumors, suggesting an increasing future role in real-world settings for the identification of patient risk groups, monitoring of patients during conventional or targeted therapies, and for the development of risk-adapted therapeutic strategies. 

## Figures and Tables

**Table 1:** Patient and sample overview.

(**A**)	
**Patient characteristics**	***n* (%)**
**Total no. of patients**	96 (100)
**Median age (years)**	66
**Female**	53 (55.2)
**Entity**	
NSCLC	56 (58.3)
Adeno	52 (54.2)
Squamous	2 (2.1)
Adeno + Squamous	1 (1.0)
NOS	1 (1.0)
Neuroendocrine carcinoma of the lung	1 (1.0)
Melanoma	31 (32.3)
CRC	3 (3.1)
Langerhans cell histiocytosis	2 (2.1)
Sarcoma	1 (1.0)
Carcinoma of the papilla of Vater	1 (1.0)
Monoclonal mast cell activation syndrome	1 (1.0)
**Stage**	
I	4 (4.2)
II	7 (7.3)
III	20 (20.8)
IV	60 (62.5)
NA	5 (5.2)
**TNM**	
T0	5 (5.2)
T1	14 (14.6)
T2	13 (13.5)
T3	18 (18.8)
T4	34 (35.4)
Tx	8 (8.3)
N0	16 (16.7)
N1	7 (7.3)
N2	32 (33.3)
N3	25 (26.0)
Nx	12 (12.5)
M0	33 (34.4)
M1	59 (61.5)
NA	4 (4.2)
(**B**)	
**Sample characteristics**	***n* (%)**
**Total no. of samples**	352 (100)
**Mean no. of samples per patient**	3.7
**ddPCR Assay**	
*EGFR* T790M	152 (43.3)
*EGFR* L858R	52 (14.6)
*EGFR* Del19	52 (14.6)
*EGFR* C797S	15 (4.3)
*EGFR* L861Q	3 (0.9)
*BRAF* V600E	44 (12.6)
*BRAF* V600K	20 (5.7)
*BRAF* V600R	2 (0.6)
*KIT* V559A	8 (2.3)
*KIT* D816V	1 (0.3)
*KRAS* G12	2 (0.6)
*NRAS* Q61R	1 (0.3)
**Mutation detection**	
Detected	107 (30.4)
Not detected	245 (69.6)
**Plasma volume used per sample**	
Median (mL)	1.75
Min (mL)	0.8
Max (mL)	17.5

Tables highlighting the most important patient (**A**) and sample (**B**) characteristics. Adeno, adenocarcinoma; CRC, colorectal carcinoma; ddPCR, digital droplet PCR; Min, minimum; Max, maximum; NA, not available; No., number; NOS, not otherwise specified; NSCLC, non-small cell lung cancer; TNM: Tumor, Nodes, Metastases.

## Supplementary Materials

Supplementary Materials are available online at https://www.mdpi.com/2075-4418/10/8/550/s1.
