# Supplementary material for: Personalized Treatment Selection and Disease Monitoring Using Circulating Tumor DNA Profiling in Real-World Cancer Patient Management
Source: Diagnostics (Basel). 2020 Aug 2;10(8):550. doi: 10.3390/diagnostics10080550 (PMC7459590; doi:10.3390/diagnostics10080550)
Supplement: Supplementary file 1 [file diagnostics-10-00550-s001.zip › Supplementary/Suppl.Table_1_Revision.pdf]

Supplementary Table S1: Detailed patient characteristics.			
**Patient_ID**	**Age**	**Sex**	**Diagnosis**
FR001	58	f	NSCLC (Adeno)
FR002	64	f	NSCLC (Adeno)
FR003	75	f	NSCLC (Adeno)
FR004	64	f	Melanoma
FR005	59	f	NSCLC (Adeno)
FR006	76	f	NSCLC (Adeno)
FR007	71	m	NSCLC (Squamous)
FR008	50	m	NSCLC (Adeno)
FR009	66	m	Melanoma
FR010	55	f	NSCLC (Adeno)
FR011	70	m	NSCLC (Adeno)
FR012	69	m	NSCLC (Adeno)
FR013	81	m	Melanoma
FR014	79	f	Melanoma
FR015	74	f	NSCLC (Adeno)
FR016	61	f	NSCLC (Adeno)
FR017	58	m	Colorectal carcinoma
FR018	68	f	NSCLC (Adeno)
FR019	73	f	NSCLC (Adeno)
FR020	68	m	NSCLC (Adeno)
FR021	63	f	NSCLC (Adeno)
FR022	60	f	NSCLC (Adeno)
FR023	60	f	NSCLC (Adeno)
FR024	77	m	Melanoma
FR025	61	f	Melanoma
FR026	76	f	NSCLC (Adeno)
FR027	55	f	Monoclonal mast cell activation syndrome
FR028	52	f	Melanoma
FR029	70	m	NSCLC (Adeno and Squamous)
FR030	78	m	NSCLC (Adeno)
FR031	73	f	NSCLC (Adeno)
FR032	72	m	NSCLC (Adeno)
FR033	79	f	Melanoma
FR034	79	f	NSCLC (Adeno)
FR035	74	m	NSCLC (Adeno)
FR036	76	m	NSCLC (Adeno)
FR037	77	m	NSCLC (Adeno)
FR038	51	f	NSCLC (Adeno)
FR039	52	f	NSCLC (Adeno)
FR040	65	m	NSCLC (Squamous)

FR041	64	m	Melanoma
FR042	54	m	Melanoma
FR043	73	f	NSCLC (Adeno)
FR044	80	m	NSCLC (Adeno)
FR045	69	m	Melanoma
FR046	74	m	NSCLC (NOS)
FR047	77	f	NSCLC (Adeno)
FR048	56	m	Colorectal carcinoma
FR049	78	f	Melanoma
FR050	44	f	Melanoma
FR051	54	m	Melanoma
FR052	82	f	Carcinoma of the papilla of Vater
FR053	54	f	Langerhans cell histiocytosis
FR054	66	m	NSCLC (Adeno)
FR055	58	m	Melanoma
FR056	61	f	Melanoma
FR057	87	f	Melanoma
FR058	54	f	NSCLC (Adeno)
FR059	51	f	NSCLC (Adeno)
FR060	63	f	Melanoma
FR061	58	f	NSCLC (Adeno)
FR062	57	f	NSCLC (Adeno)
FR063	63	m	NSCLC (Adeno)
FR064	77	m	Melanoma
FR065	27	m	Choroidal Melanoma
FR066	60	m	NSCLC (Adeno)
FR067	59	f	Neuroendocrine Carcinoma of the Lung
FR068	71	m	NSCLC (Adeno)
FR069	68	f	NSCLC (Adeno)
FR070	60	f	NSCLC (Adeno)
FR071	62	f	NSCLC (Adeno)
FR072	43	f	Melanoma
FR073	73	m	NSCLC (Adeno)
FR074	55	m	Melanoma
FR075	67	f	Melanoma
FR076	72	f	NSCLC (Adeno)
FR077	75	m	Melanoma
FR078	66	m	NSCLC (Adeno)
FR079	40	f	Colorectal carcinoma
FR080	60	m	Melanoma
FR081	49	m	Melanoma
FR082	51	m	Melanoma
FR083	59	f	NSCLC (Adeno)

FR084	57	m	NSCLC (Adeno)
FR085	76	f	NSCLC (Adeno)
FR086	69	f	NSCLC (Adeno)
FR087	63	f	NSCLC (Adeno)
FR088	58	m	Melanoma
FR089	91	f	Melanoma
FR090	78	m	Melanoma
FR091	68	m	Sarcoma
FR092	71	f	Melanoma
FR093	60	f	NSCLC (Adeno)
FR094	79	m	Langerhans cell histiocytosis
FR095	79	m	NSCLC (Adeno)
FR096	79	f	NSCLC (Adeno)

NSCLC = Non-small cell lung cancer
Adeno = Adenocarcinoma
Squamous = Squamous cell carcinoma
f = female
m = male

**Initial stage**	**TNM**	**No. ddPCR tests ordered**	
IV	cT3 cN0 cM1	2	
IV	cT2a pN2 cM0	12	
IV	cT3 cN3 cM1b	2	
IV	Tx Nx M1c	1	
IV	cT4 cN3 cM1a	8	
IV	cT4 cN3 cM1a	3	
IIIA	cT4 cN1 cM0	1	
IV	cT2b cN3 cM1c	5	
IIID	pT3b cN3c cM0	2	
IIIA	pT1b pN2 cM0	1	
IV	cT4 cN3 cM1b	2	
IV	T3 Nx M1	53	
IV	pT1a cN2b M1c	2	
III	Tx pN1b cM0	2	
IV	cT4 cN3 cM1b	1	
IV	cT4 cN3 cM1	1	
IV	pT4b pN2b pM1	3	
IV	cT1b cN2 cM1b	14	
IV	cT2a cN3 cM1	4	
IV	cT4 cN3 cM1b	2	
IV	T3 N2 M1	8	
IV	cT4 cN3 cM1a	8	
IIA	pT3b pN0 cM0	5	
IV	cT0 cNx pM1c	1	
IV	Tx Nx M1	2	
IB	pT2a pN0 pM0	4	
NA	NA	1	
IIID	pT4b pN3 M1	10	
IIIA	pT1 pN2 cM0	8	
IV	cT4 cN0 cM1a	3	
IV	cT4 cN3 cM1c	8	
IV	cT2a cN3 cM1	3	
IV	pT4a cN0 M1	2	
IIIA	pT1 pN2 cM0	1	
IV	cT2 cN3 cM1c	5	
NA	NA	2	
IV	pT4 cN3 pM1b	1	
IIIA	pT1b pN2 cM0	1	
IVA	cT2 cN2 cM1a	1	
IV	cT4 cN3 cM1	1	

IV	pT1b Nx M1c	1	
IV	pT1b pNx pM1d	1	
IV	cT4 cN2 cM1c	4	
IIA	pT2 pN0 pM0	2	
IIIB	T0 N1c cM0	3	
IV	cT4 pN2 cM1b	2	
IV	pT3 pN2 pM1a	4	
IIA	pT3 pN1b cM0	4	
IV	T0 Nx M1c	2	
IV	T0 cN3c cM0	2	
IIA	pT2b N0 M0	1	
I	pT1 N0 M0	1	
NA	NA	1	
II	pT3 cN0 cM0	2	
IIIC	pT4b pN2 cM0	1	
IV	pT3b N2c M1	1	
IV	pTx cNx cM1	2	
IA	pT1b pN0 cM0	3	
IIIA	cT3 cN2 cM0	2	
IIB	pT4 N0 M0	1	
IIIA	pT1a pN2 cM0	4	
IV	cT4 cN3 cM1b	1	
IIB	pT3 pN0 cM0	2	
IA	pT1b pN0 cM0	2	
NA	Tx N0 M0	1	
IV	cT2a cN2 cM1b	21	
IIIC	cT4 cN2 cM0	2	
IV	Tx Nx M1	1	
IV	cT3 cN2 pM1a	7	
IV	cT2a cN2 cM1b	15	
IV	pT4 cN3 cM1b	4	
IV	pT4b pN1 cM1c	1	
IV	cT4 cN3 cM1	5	
IIIB	pT4b pN2a cM0	1	
IV	pT1a pN2b pM1c	1	
IV	cT4 cN0 pM1a	1	
IIIC	pT3b pN1a cM0	2	
IV	pT4 pN2 pM1a	6	
IV	ypT2 pN2 pM1	1	
IV	Tx Nx M1c	2	
IIID	pT4b pN3b cM0	2	
IIIC	pT3b pN1a cM0	2	
IV	cT4 cN2 cM1c	6	

IVA	pT4 pN2 cM1b	1	
IV	T3 N3 M1	2	
IIIA	pT2a pN2 cM0	4	
IV	pT3 pN2 cM1b	1	
IV	pT0 pN3 cM1	1	
IV	pT4b cN0 M1c	2	
IV	pT4b Nx M1	1	
IIIB	pT3 Nx M0	1	
IIIC	pT4b pN2a cM0	2	
IVA	cTx pN2 cM1a	3	
NA	NA	1	
IV	cT4 pN3 cM1c	4	
IV	cT1c cN2 cM1a	3	
	**Average**	**3.7**	

