# Supplementary material for: Personalized Treatment Selection and Disease Monitoring Using Circulating Tumor DNA Profiling in Real-World Cancer Patient Management
Source: Diagnostics (Basel). 2020 Aug 2;10(8):550. doi: 10.3390/diagnostics10080550 (PMC7459590; doi:10.3390/diagnostics10080550)
Supplement: Supplementary file 1 [file diagnostics-10-00550-s001.zip › Supplementary/Suppl.Table_2_Revision.pdf]

**Supplementary Table S2: Overview ddPCR assay development ar**			
**Gene**	**AA change**	**LOD (MUT:WT)**	**LOD (%)**
EGFR	T790M	1:1122	0.089
EGFR	del19	1:753	0.130
EGFR	L858R	1:1316	0.076
EGFR	C797S	1:2140	0.047
EGFR	L861Q	1:2584	0.039
BRAF	V600E	1:2145	0.047
BRAF	V600K	1:6530	0.015
BRAF	V600R	1:2019	0.050
*KRAS**	G12V	1:9041	0.011
NRAS	Q61R	1:994	0.010
KIT	V559A	1:1396	0.072
KIT	D816V	1:2174	0.046
* This assay was developed by Hussung et al., J Mol Diagn, 2020, PM			

nd validation.		
**LOB (MUT copies/mL plasma)**	**Specificity**	
3.2	100%	
17.8	100%	
2.7	100%	
1.7	100%	
1.7	100%	
5.9	100%	
3.1	100%	
3.3	100%	
2.2	100%	
6.2	100%	
2.7	100%	
0	100%	
IID: 32376474		
