# Supplementary material for: Personalized Treatment Selection and Disease Monitoring Using Circulating Tumor DNA Profiling in Real-World Cancer Patient Management
Source: Diagnostics (Basel). 2020 Aug 2;10(8):550. doi: 10.3390/diagnostics10080550 (PMC7459590; doi:10.3390/diagnostics10080550)
Supplement: Supplementary file 1 [file diagnostics-10-00550-s001.zip › Supplementary/Supplementary_figures.pdf]

Supplementary data for

Personalized Treatment Selection and Disease Monitoring by Circulating Tumor DNA Profiling in Real-World Cancer Patient Management

Julius Wehrle, Ulrike Philipp, Martina Jolic, Marie Follo, Saskia Hussung, Silvia Waldeck, Max Deuter, Michael Rassner, Jan Braune, Justyna Rawluk, Christine Greil, Cornelius F. Waller, Heiko Becker, Jesús Duque-Afonso, Anna-L. Illert, Ralph M. Fritsch, Frank Meiss, Justus Duyster, Nikolas von Bubnoff[#]*, Florian Scherer[#]*

[#]Equal contribution
*Corresponding authors. E-mails: florian.scherer@uniklinik-freiburg.de; nikolas.vonbubnoff@uksh.de

The file includes:
Suppl. Fig. S1. Specifications of digital droplet PCR assay development.
Suppl. Fig. S2. Digital droplet PCR assay development.
Suppl. Fig. S3. Digital droplet PCR assay validation.
Suppl. Fig. S4. Digital droplet PCR performance.
Suppl. Fig. S5. Prediction and detection of disease progression by ctDNA monitoring.
Suppl. Fig. S6. Identification of actionable gene targets by ctDNA profiling.
Suppl. Fig. S7. Detection of resistance mutations by noninvasive ctDNA assessment.
Suppl. Table S1. Detailed patient characteristics.
Suppl. Table S2. Overview ddPCR assay development and validation.

A

EGFR **del19**			
**Primer**			
**Gene**	Target	Direction	Sequence
EGFR	Ex.19del	forward	5'- CCA GAA GGT GAG AAA GTT A-3'
		reverse	5'- CAG CAA AGC AGA AAC TCA-3'
**Probes**			
**Gene**	Target	Mutation	Sequence
EGFR	Ex.19del	Reference LNA	5'- /5HEX/CAA+CAA+GGA+AAT+CCT CGA T /3IABkFQ/ -3'
		Hotspot LNA	5'- /56-FAM/TGC+TTC+TCT+TAA+TTC CTT G /3IABkFQ/ -3'

EGFR **T790M**			
**Primer**			
**Gene**	Target	Direction	Sequence
EGFR	T790M	forward	5'- CAG GAA GCC TAC GTG ATG-3'
		reverse	5'- GTG TTC CCG GAC ATA GTC-3'
**Probes**			
**Gene**	Target	Mutation	Sequence
EGFR	T790M	wild type	5'- /5HEX/C ATG+AGC+TGC+GTG+ATG AGC/3IABkFQ/ -3'

		c.2369C>T	5'- /56-FAM/C ATG+AGC+TGC+ATG+ATG AGC/3IABkFQ/ -3'

EGFR **L858R**			
**Primer**			
**Gene**	Target	Direction	Sequence
EGFR	L858R	forward	Bio-Rad Laboratories, Inc. (UniqueAssayID: dHsaMDV2010021)
		reverse	Bio-Rad Laboratories, Inc. (UniqueAssayID: dHsaMDV2010021)
**Probes**			
**Gene**	Target	Mutation	Sequence
EGFR	L858R	wild type	Bio-Rad Laboratories, Inc. (UniqueAssayID: dHsaMDV2010021)
		c.2573T>G	Bio-Rad Laboratories, Inc. (UniqueAssayID: dHsaMDV2010021)

EGFR **C797S**			
**Primer**			
**Gene**	Target	Direction	Sequence
EGFR	C797S	forward	5'- CAC CGT GCA GCT CAT CAC -3'
		reverse	5'- CAC ACC AGT TGA GCA GGT A -3'
**Probes**			
**Gene**	Target	Mutation	Sequence
EGFR	C797S	wild type	5'- /5HEX/ T TCG+GCT+GCC+TCC+TGG A /3IABkFQ/ -3'
		c.2390G>C	5'- /56-FAM/ T TCG+GCT+CCC+TCC+TGG A /3IABkFQ/ -3'

EGFR **L861Q**			
**Primer**			
**Gene**	Target	Direction	Sequence
EGFR	L861Q	forward	Bio-Rad Laboratories, Inc. (UniqueAssayID: dHsaMDV2010043)
		reverse	Bio-Rad Laboratories, Inc. (UniqueAssayID: dHsaMDV2010043)
**Probes**			
**Gene**	Target	Mutation	Sequence
EGFR	L861Q	wild type	Bio-Rad Laboratories, Inc. (UniqueAssayID: dHsaMDV2010043)
		c.2582T>A	Bio-Rad Laboratories, Inc. (UniqueAssayID: dHsaMDV2010043)

BRAF **V600E**			
**Primer**			
**Gene**	Target	Direction	Sequence
BRAF	V600E	forward	5'- GAT CCA GAC AAC TGT TCA -3'
		reverse	5'- ACA CCT CAG ATA TAT TTC TTC A -3'
**Probes**			
**Gene**	Target	Mutation	Sequence
BRAF	V600E	wild type	5'- /5HEX/ T CGA+GAT+TTC+ACT+GTA GCT /3IABkFQ/ -3'
		c.1799T>A	5'- /56-FAM/ T CGA+GAT+TTC+TCT+GTA GCT /3IABkFQ/ -3'

BRAF **V600K**			
**Primer**			
**Gene**	Target	Direction	Sequence
BRAF	V600K	forward	5'- CAC CTC AGA TAT ATT TCT TCA TG-3'
		reverse	5'- GAT CCA GAC AAC TGT TCA A-3'

**Probes**			
**Gene**	Target	Mutation	Sequence
BRAF	V600K	wild type	5'- /5HEX/ CTA+GCT+ACA+GTG+AAA TCT C /3IABkFQ/ -3'
		c.1798_1799delinsAA	5'- /56-FAM/ TAG+CTA+CAA+AGA+AAT CTC G/3IABkFQ/-3'

***BRAF* V600R**			
**Primer**			
**Gene**	Target	Direction	Sequence
BRAF	V600R	forward	5'- ACA CCT CAG ATA TAT TTC TTC A-3'
		reverse	5'- CCA GAC AAC TGT TCA AAC-3'
**Probes**			
**Gene**	Target	Mutation	Sequence
BRAF	V600R	wild type	5'- /5HEX/ C GAG+ATT+TCA+CTG+TAG C/3IABkFQ/ -3'
		c.1798_1799delinsAG	5'- /56-FAM/ C GAG+ATT+TCC+TTG+TAG C/3IABkFQ/-3'

***KRAS* G12V**			
**Primer**			
**Gene**	Target	Direction	Sequence
KRAS	G12V	forward	5'- GCC TGC TGA AAA TGA CTG-3'
		reverse	5'- GCT GTA TCG TCA AGG CAC-3'
**Probes**			
**Gene**	Target	Mutation	Sequence
KRAS	G12V	wild type	5'- /5HEX/ CC+A +C+C+A G+CT C/3IABkFQ/ -3'
		c.35G>A	5'- /56-FAM/ CG+C C+A+A +CAG +CT/3IABkFQ/-3'

***NRAS* Q61R**			
**Primer**			
**Gene**	Target	Direction	Sequence
NRAS	Q61R	forward	5'- TCC TCA TGT ATT GGT CTC-3'
		reverse	5'- AGT GGT TAT AGA TGG TGA A-3'
**Probes**			
**Gene**	Target	Mutation	Sequence
NRAS	Q61R	wild type	5'- /5HEX/ CT CTT+CT+TG+TC+CAG C/3IABkFQ/ -3'
		c.182A>G	5'- /56-FAM/ CT CTT+CT+CG+TC+CAG C/3IABkFQ/-3'

***KIT* V559A**			
**Primer**			
**Gene**	Target	Direction	Sequence
KIT	V559A	forward	5'- CAC AGA AAC CCA TGT ATG-3'
		reverse	5'- GTT GGG TCT ATG TAA ACA TAA-3'
**Probes**			
**Gene**	Target	Mutation	Sequence
KIT	V559A	wild type	5'- /5HEX/ CCT CAA+CAA+CCT+TCC+ACT/3IABkFQ/ -3'
		c.1676T>C	5'- /56-FAM/ CCT CAA+CAG+CCT+TCC+ACT/3IABkFQ/-3'

***KIT* D816V**
**Primer**

Gene	Target	Direction	Sequence
KIT	D816V	forward	5'- CAT GGT CGG ATC ACA AAG-3'
		reverse	5'- CTG TCA AGC AGA GAA TGG-3'
**Probes**			
**Gene**	Target	Mutation	Sequence
KIT	D816V	wild type	5'- /5HEX/ TAG CCA+GAG+ACA+TCA+AGA A/3IABkFQ/ -3'
		c.2447A>T	5'- /56-FAM/ TAG CCA+GAG+TCA+TCA+AGA AT/3IABkFQ/-3'

B

Cycling Step	Temperature, °C	Time	Number of Cycles
**Enzyme activation**	95	10 min	1
**Denaturation**	95	30 sec	40 (*EGFR*del, *EGFR* T790M, *EGFR* L858R, *EGFR* C797S, *EGFR* L861Q, *BRAF* V600E, *BRAF* V600K, *BRAF* V600R, *KRAS* G12V, *KIT* V559A, *KIT* D816V)<br>50 (*NRAS* Q61R)
**Annealing/ Extension**	55.0 (*EGFR*del, *EGFR* L858R, *EGFR* L861Q, *BRAF* V600K, *BRAF* V600R)<br>56.8 (*NRAS* Q61R)<br>57 (*BRAF* V600E)<br>58 (*KIT* D816V)<br>58.5 (*KIT* V559A)<br>59.4 (*KRAS* G12V)<br>63 (*EGFR* T790M, *EGFR* C797S)	60 sec (*EGFR*del, *EGFR* T790M, *EGFR* L858R, *EGFR* C797S, *EGFR* L861Q, *KIT* V559A)<br>90 sec (*BRAF* V600E, *BRAF* V600K, *BRAF* V600R, *KRAS* G12V, *NRAS* Q61R, *KIT* D816V)	
**Enzyme deactivation**	98	10 min	1
**Hold (optional)**	12	Infinite	1

Suppl. Fig. S1. Specifications of digital droplet PCR assay development. Shown are primer/probe sequences (A) and cycling conditions (B) for all ddPCR assays. Primers and probes for the *EGFR* L858R and L861Q assays were obtained from Bio-Rad Laboratories. The *KRAS* G12V assay was developed by Hussung et al., J Mol Diagn., 2020, PMID: 32376474.

A

Dilution – Expected AF (%)	Ø MUT copies/well	Ø WT copies/well	MUT:WT	SD	Detected AF (%)	Significance
NTC	0	0				
WT	5.05	18710		2	0.027	
5%	1035.5	19015	1:18	28	5.45	***
2,50%	528.5	19015	1:36	24	2.78	***
1%	207.5	18915	1:91	8	1.1	***
0,50%	98.5	18845	1:191	13	0.52	***
0,25%	52	18925	1:364	3	0.27	***
0,125%	29.35	18620	1:634	5	0.16	***
0,0975%	25.2	18210	1:723	3	0.14	***
0,05%	16.75	18798	1:1122	4	0.089	***
0,01%	8.03	18110	1:2255	3	0.044	n.s.

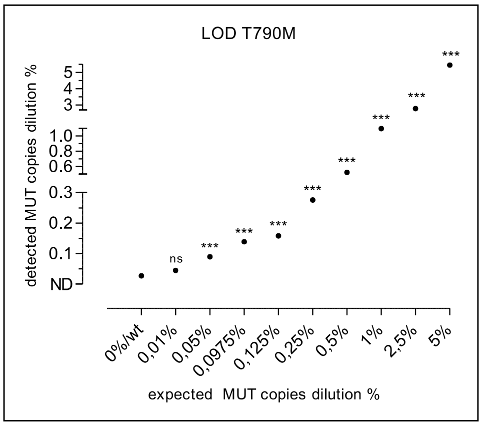

B

Sample	Vol. plasma (mL)	Vol. eluate (µl)	mL plasma / well	Ø WT copies/ well	Ø WT copies /mL plasma	Ø MUT copies /well	Ø MUT copies /mL plasma
NTC				0		0	
WT				850		1	
MIX				2680		607	
HC 1	1.8	67	0.19	220	1170	0	0
HC 2	1.9	65	0.20	262	1280	0.65	3.2
HC 3	1.85	72	0.18	220	1223	0	0
HC 4	1.95	71	0.19	206	1072	0	0
HC 5	2	74	0.19	182	962	0	0
HC 6	1.98	63	0.22	87	395	0	0
HC 7	1.9	65	0.20	156	762	0	0
HC 8	1.95	73	0.19	476	2546	0	0
HC 9	2	65	0.22	153	708	0	0
HC 10	2	66	0.21	113	533	0	0
Mean						0.1	0.3
σ						0.195	0.953
**LOB *EGFR* T790M**						**0.7**	**3.2**

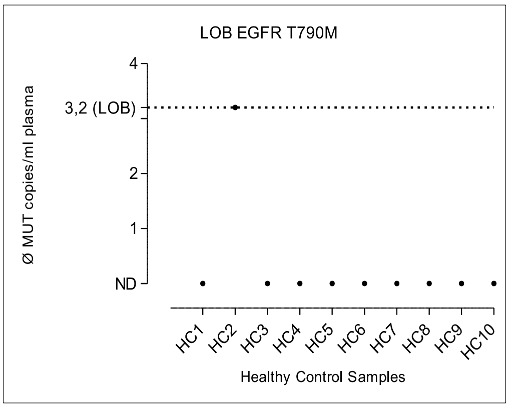

Suppl. Fig. S2. Digital droplet PCR assay development. Design and development of the *EGFR* T790M digital droplet PCR (ddPCR) assay, representative of all the assays developed in this work. (A) Left: Specifications and results of the dilution series performed to assess limit of detection (LOD), using recombinant mutant DNA fragments in the presence of human genomic DNA. LOD was determined as the ratio of mutant vs. total number of copies from the highest dilution that was significantly above the background, in this case 1:1122 copies/well (= 0.089%). Right: Scatter plot showing the correlation of expected mutant copies (in %) with detected mutant copies (in %) in the dilution series. (B) Determining limit of blank (LOB) by analyzing cell-free DNA (cfDNA) extracted from 10 healthy controls. Left: Table showing plasma/cfDNA input information and test results, with 3.2 mutant copies per mL of plasma being the LOB calculated for the *EGFR* T790M assay (see Materials and Methods). Right: Number of positive mutant copies per mL of plasma in each healthy control sample (HC1 – HC10). Red dashed line: LOB. LODs and LOBs of all assays are depicted in Suppl. Table 2. AF, allele frequency; Vol., volume; MUT, mutant; WT, wildtype; SD (σ), standard deviation; mL, milliliters; µl, microliters; NTC, non-template control; HC, healthy control.

Sample	Vol. plasma (mL)	Vol. eluate (µl)	mL plasma/ well	Ø MUT copies/well	Ø WT copies/well	Ø MUT copies/ mL plasma	Ø WT copies/ mL plasma	AF [%]
LBL-024	1.95	75	0.18	0	127	0	697.8	0
LBL-025	1.7	73	0.16	0	148	0	907.9	0
LBL-026	1.7	74	0.16	0	349	0	2170.3	0
LBL-027	1.65	66	0.18	0	52	0	297.1	0
LBL-028	1.7	64	0.19	0	112	0	602.4	0
LBL-029	1.65	65	0.18	0.3	130	1.7	731.6	0.23
LBL-030	1.6	65	0.17	0	119.5	0	693.5	0
LBL-031	1.55	66	0.16	0	150.5	0	915.5	0
LBL-032	1.75	71	0.17	0	138	0	799.8	0
LBL-033	1.9	67	0.199	0	246.5	0	1241.8	0

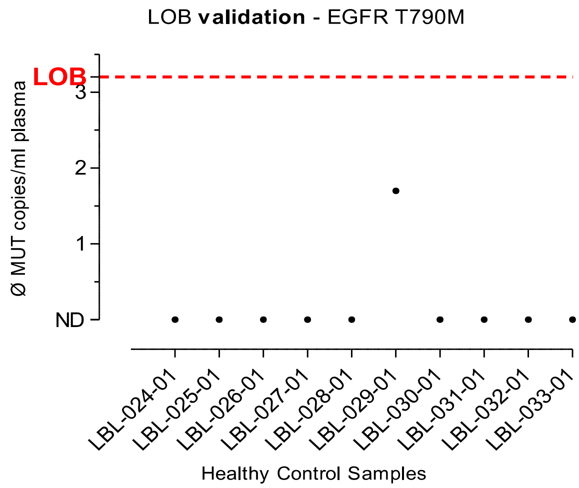

Suppl. Fig. S3. Digital droplet PCR assay validation. Validation of the *EGFR* T790M ddPCR assay, representative of all the assays developed in this work. Specificity was assessed by running the *EGFR* T790M assay on cfDNA obtained from an independent cohort of 10 healthy donors. Top: Table showing plasma/cfDNA input information and test results. Bottom: Number of positive mutant copies per mL of plasma in each healthy control sample (LBL-024 – LBL-033). Dashed red line: LOB defined in Suppl. Figure 1. AF, allele frequency; Vol., volume; MUT, mutant; WT, wildtype; mL, milliliters; LOB, limit of blank.

A

	Tumor positive	Tumor negative	**Total (*n*)**	**Performance**
Plasma positive	14	2*	**16**	**PPV: 87.5% (*100%)**
Plasma negative	2	38	**40**	**NPV: 95%**
**Total (*n*)**	**16**	**40**	**56**	
**Performance**	**Sensitivity: 87.5%**	**Specificity: 95% (*100%)**		

*** Tumor results likely false negative**

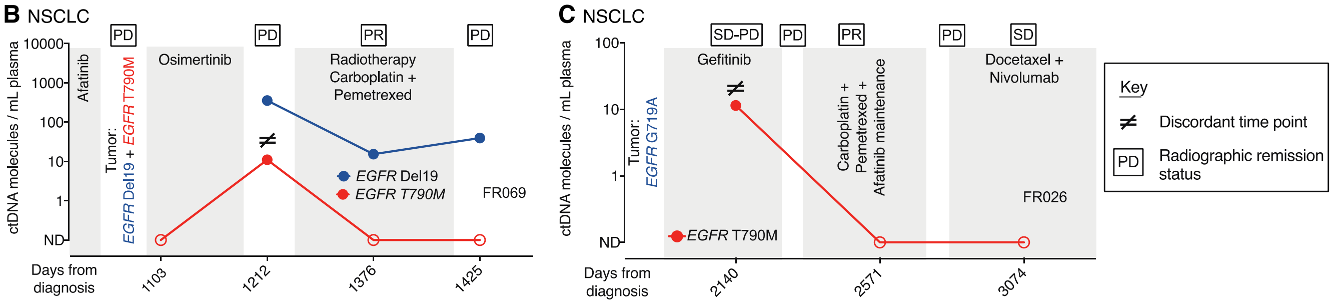

Suppl. Fig. S4. Digital droplet PCR performance. (A) Table showing sensitivity, specificity, positive predictive value, and negative predictive value of all ddPCR assays developed in this work compared to tumor tissue genotyping as a gold standard. Asterisk: 2 plasma samples showed *EGFR* T790M mutations while genotyping from tumor biopsies did not identify these aberrations. (B, C) Shown are the concentrations of ctDNA molecules (*EGFR* Del19 and *EGFR* T790M) per mL of plasma in serial blood samples obtained from the two NSCLC patients with discordant results of matched tumor-plasma samples. Crossed equal signs represent discordant time points when ctDNA was detected in plasma but mutations were not found in tumor biopsies. PPV, positive predictive value; NPV, negative predictive value; PD, progressive disease; PR, partial response; SD, stable disease; mL, milliliters; ND, not detected; NSCLC, non-small cell lung cancer.

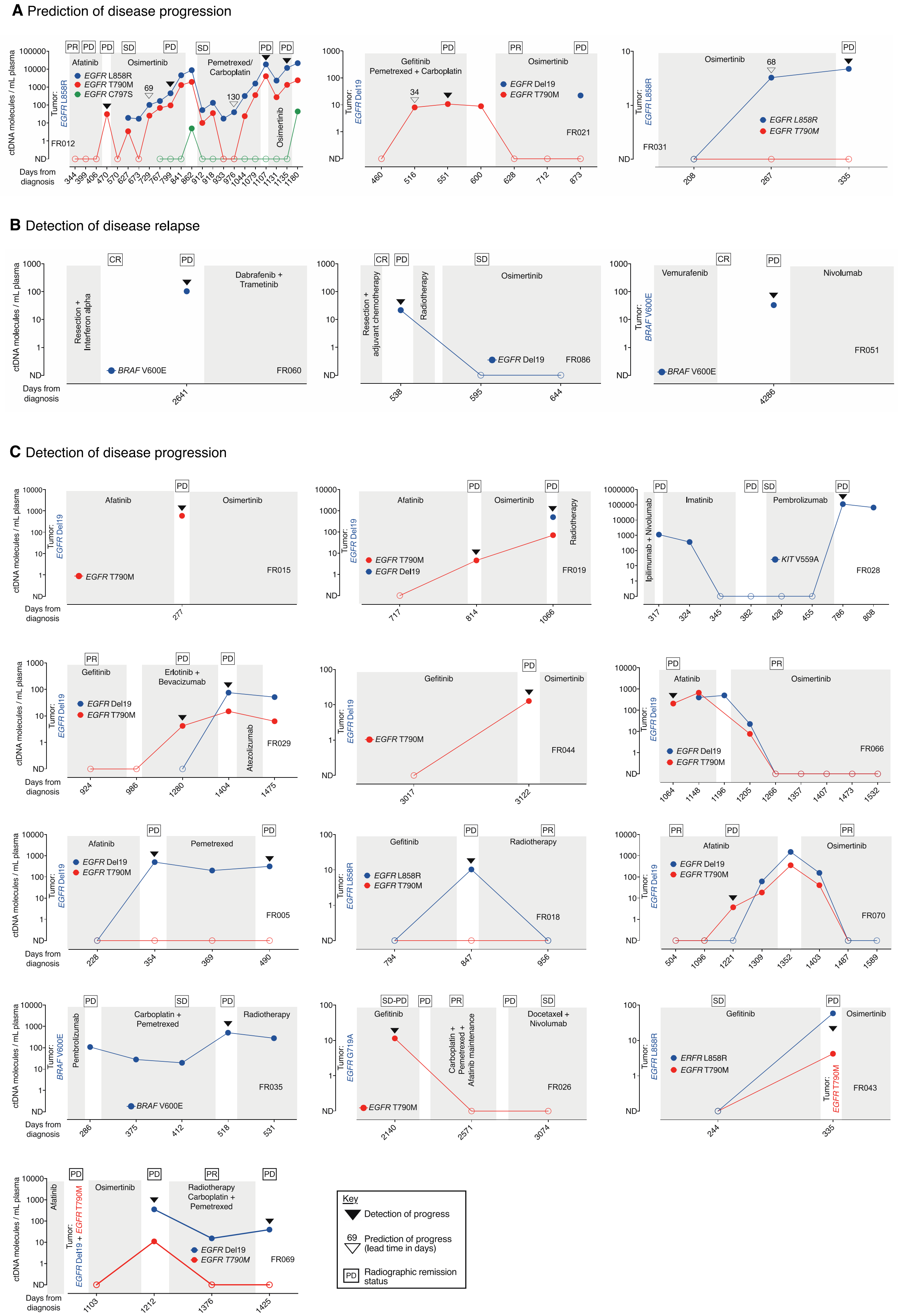
A Prediction of disease progression
B Detection of disease relapse
C Detection of disease progression
ctDNA molecules / mL plasma
Days from diagnosis
FR012
FR021
FR031
FR060
FR086
FR051
FR015
FR019
FR028
FR029
FR044
FR066
FR005
FR018
FR070
FR035
FR026
FR043
FR069
Key
Detection of progress
69 Prediction of progress (lead time in days)
PD Radiographic remission status

Suppl. Fig. S5. Prediction and detection of disease progression by ctDNA monitoring. Serial monitoring of blood plasma highlighting the value of ctDNA as a biomarker for prediction (A) and detection (B, C) of disease progression or relapse after complete response, either by an increase of ctDNA concentrations over time or the identification of new aberrations reflecting emerging subclones. Triangles highlight plasma sample with increasing ctDNA levels in plasma, either predicting (empty triangles) or detecting radiographic disease progression (black triangles). Numbers above empty triangles represent the time from increase of ctDNA concentrations to radiographic disease progression in days. Tumor genotyping results at diagnosis are depicted on the left side of each graph. Black rectangle, radiographic remission status; PD, progressive disease; PR, partial response; SD, stable disease; CR, complete response; mL, milliliters; ND, not detected.

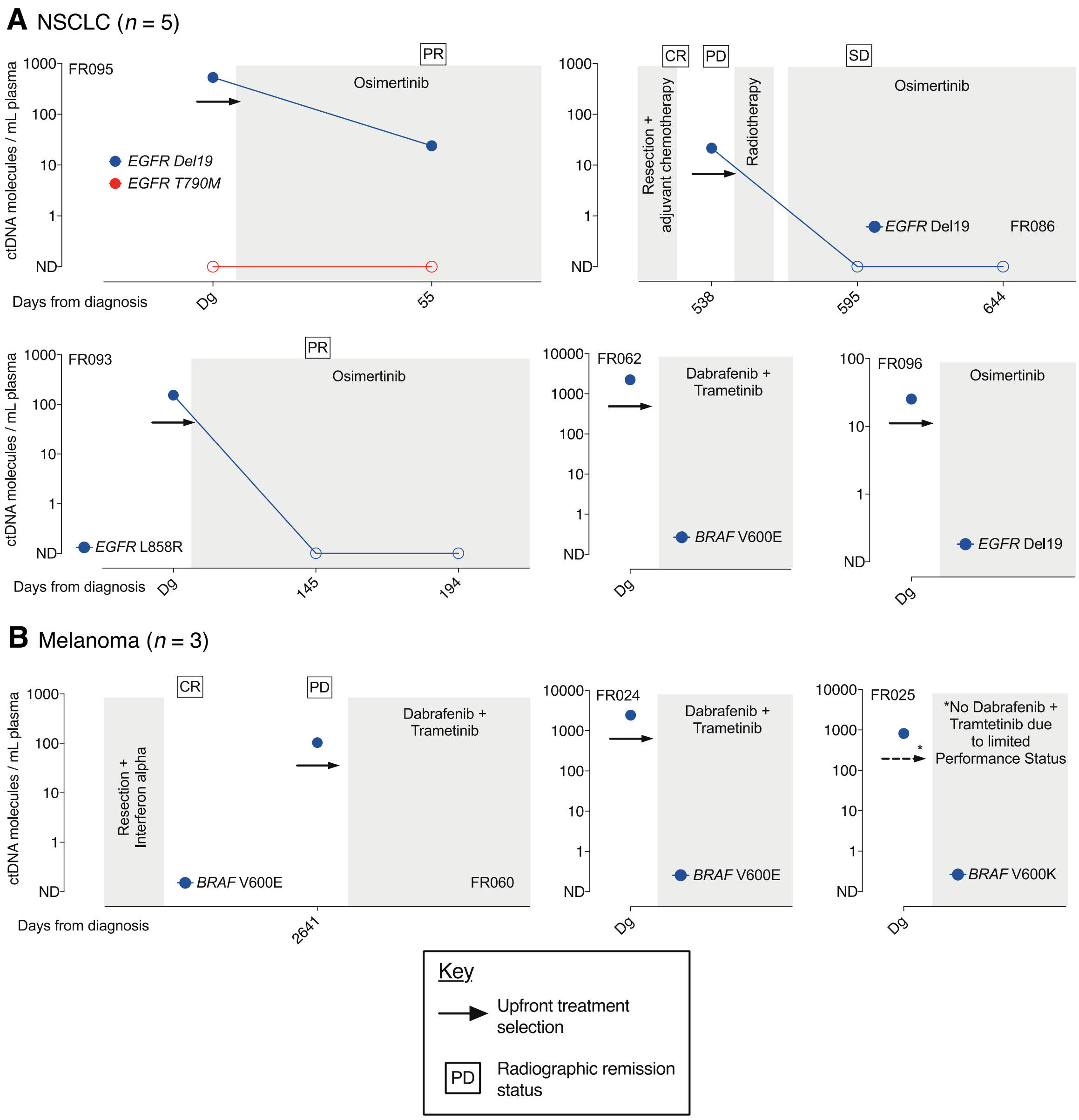

Suppl. Fig. S6. Identification of actionable gene targets by ctDNA profiling. Shown are five NSCLC cases (A) and three melanoma patients (B) in whom detection of tumor

mutations in druggable gene targets led to targeted treatment selection and in whom these mutations were also detected in plasma ctDNA. Black arrows highlight treatment selection based on mutation detection in druggable gene targets. Black rectangle, radiographic remission status; PD, progressive disease; PR, partial response; SD, stable disease; CR, complete response; mL, milliliters; ND, not detected; NSCLC, non-small cell lung cancer.

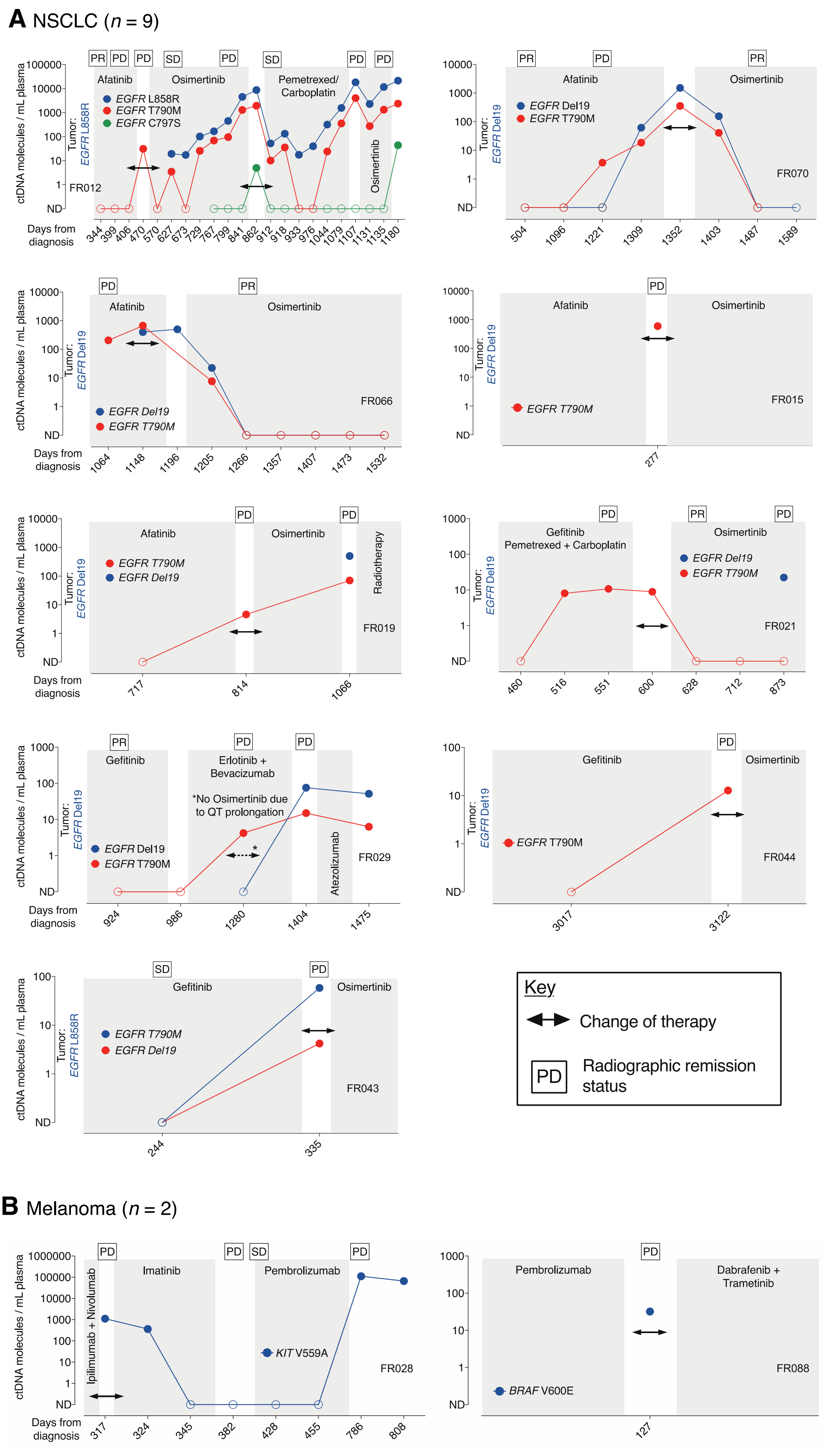
A NSCLC (n = 9)
ctDNA molecules / mL plasma
Days from diagnosis
FR012
Tumor: EGFR L858R
PR PD PD SD PD SD PD PD
Afatinib
Osimertinib
Pemetrexed/ Carboplatin
Osimertinib
EGFR L858R
EGFR T790M
EGFR C797S
344 399 406 470 570 627 673 729 767 799 841 862 912 918 933 976 1044 1079 1107 1131 1135 1180
FR070
Tumor: EGFR Del19
PR PD PR
Afatinib
Osimertinib
EGFR Del19
EGFR T790M
504 1096 1221 1309 1352 1403 1487 1589
FR066
Tumor: EGFR Del19
PD PR
Afatinib
Osimertinib
EGFR Del19
EGFR T790M
1064 1148 1196 1205 1266 1357 1407 1473 1532
FR015
Tumor: EGFR Del19
PD
Afatinib
Osimertinib
EGFR T790M
277
FR019
Tumor: EGFR Del19
PD PD
Afatinib
Osimertinib
Radiotherapy
EGFR T790M
EGFR Del19
717 814 1066
FR021
Tumor: EGFR Del19
PD PR PD
Gefitinib
Pemetrexed + Carboplatin
Osimertinib
EGFR Del19
EGFR T790M
460 516 551 600 628 712 873
FR029
Tumor: EGFR Del19
PR PD PD
Gefitinib
Erlotinib + Bevacizumab
*No Osimertinib due to QT prolongation
Atezolizumab
EGFR Del19
EGFR T790M
924 986 1280 1404 1475
FR044
Tumor: EGFR Del19
PD
Gefitinib
Osimertinib
EGFR T790M
3017 3122
FR043
Tumor: EGFR L858R
SD PD
Gefitinib
Osimertinib
EGFR T790M
EGFR Del19
244 335
Key
Change of therapy
PD Radiographic remission status
B Melanoma (n = 2)
FR028
PD PD SD PD
Ipilimumab + Nivolumab
Imatinib
Pembrolizumab
KIT V559A
317 324 345 382 428 455 786 808
FR088
PD
Pembrolizumab
Dabrafenib + Trametinib
BRAF V600E
127

Suppl. Fig. S7. Detection of resistance mutations by noninvasive ctDNA assessment. This figure demonstrates 9 NSCLC (A) and 2 melanoma patients (B) in whom treatment was adapted based on the detection of novel or resistance mutations and in whom these aberrations were also identified in plasma ctDNA. Black arrows highlight the time point of mutation detection that led to treatment adjustment. Tumor genotyping results at diagnosis are depicted on the left side of each graph. Black rectangle, radiographic remission status; PD, progressive disease; PR, partial response; SD, stable disease; CR, complete response; mL, milliliters; ND, not detected; NSCLC, non-small cell lung cancer.
